# Elevated inflammatory biomarkers during unemployment: modification by age and country in the UK

**DOI:** 10.1136/jech-2014-204404

**Published:** 2015-02-19

**Authors:** Amanda Hughes, Anne McMunn, Mel Bartley, Meena Kumari

**Affiliations:** 1 Department of Epidemiology and Public Health, University College London, London, UK; 2 Institute for Social and Economic Research, University of Essex, Colchester, UK

**Keywords:** UNEMPLOYMENT, Social and life-course epidemiology, Molecular Epidemiology

## Abstract

**Background:**

There is raised risk of mortality following unemployment, and reviews have consistently found worse psychological health among the unemployed. Inflammation is increasingly implicated as a mediating factor relating stress to physical disease and is strongly linked to depression. Inflammation may, therefore, be implicated in processes associated with excess mortality and morbidity during unemployment. This study examined associations of unemployment with inflammatory markers among working-age men and women from England and Scotland.

**Methods:**

Cross-sectional analyses using data from the Health Survey for England and the Scottish Health Survey collected between 1998 and 2010. Systemic inflammation was indexed by serum concentrations of C reactive protein (CRP) and fibrinogen, and compared between participants currently employed/self-employed, currently unemployed and other groups.

**Results:**

CRP, fibrinogen and odds of CRP >3 mg/L were all significantly raised for the unemployed, as compared to the employed participants (eg, OR for CRP >3 mg/L=1.43, CI 1.15 to 1.78 N=23 025), following adjustment for age, gender, occupational social class, housing tenure, smoking, alcohol consumption, body mass index, long-term illness and depressive/anxiety symptoms. Strengths of associations varied considerably by both age and country/region, with effects mainly driven by participants aged ≥48 and participants from Scotland, which had comparatively high unemployment during this time.

**Conclusions:**

Current unemployment is associated with elevated inflammatory markers using data from two large-scale, nationally representative UK studies. Effect modification by age suggests inflammation may be particularly involved in processes leading to ill-health among the older unemployed. Country/regional effects may suggest the relationship of unemployment with inflammation is strongly influenced by contextual factors, and/or reflect life course accumulation processes.

## Introduction

There is raised risk of mortality following unemployment^1–3^ and reviews have consistently found worse psychological health among the unemployed.^4–6^ Unemployment is a stressful life event, often involving loss not only of financial resources but psychosocial assets, such as time structure, status and social support.^7^ The inflammatory response occurs in response to infection or injury, where it helps to fight infection and repair damaged tissue,^8^ but can also occur for extended periods of time in the absence of infection or injury. Such ‘systemic’ inflammation is increasingly implicated as a mediating factor relating stress to cardiovascular disease^9^ and is strongly linked to depression.^10^ It is, therefore, plausible that inflammatory markers may be elevated in the unemployed, and reflect processes associated with the excess morbidity and mortality in this group. To our knowledge, two small-scale studies have examined associations of unemployment with inflammatory markers. We explored this association in a large data set of 23 025 participants from the Health Survey for England (HSE) and Scottish Health Survey (SHeS), allowing for a wide range of potential confounders and mediators to be explored.

## Methods

### Participants

The HSE and the SHeS are annual government surveys, each comprising a new sample every year, with core samples nationally representative of residents with private addresses.^11^ ^12^ Each has a stratified two-stage sampling design, with households selected from primary sampling units.^13^ This analysis was restricted to core-sample participants of working age, defined as 16–64 last birthday.

Surveys consisted of a face-to-face interview followed by a nurse visit during which clinical measurements were taken, including serum C reactive protein (CRP) and fibrinogen, both markers of systemic inflammation. Data was aggregated from nine HSE and SHeS surveys in which CRP and fibrinogen measurements were taken for the core sample: HSE 1998, 1999, 2003, 2006 and 2009, and SHeS 2003, 2008, 2009 and 2010. From HSE 1999 and from the 2008 SHeS only a subsample of core sample adults were targeted for a nurse visit; so only these participants had measurements of CRP and fibrinogen. Observations from SHeS 2011 were not used, because introduction of a different CRP analyser resulted in measured CRP concentrations on average 15 mmol/L higher, leading to concerns about consistency.^14^

The initial sample comprised all core sample working-age adults from nine surveys targeted for a blood sample (N=49 385). Of these, 43 129 (87.3%) consented to a nurse visit but only 30 103 (61%) consented to a blood sample. Problems in taking samples, laboratory problems with samples obtained, and non-measurement of fibrinogen for participants taking fibrates resulted in 27 366 CRP measurements and 24 551 fibrinogen measurements.

Participants with CRP >10 mg/L were excluded from CRP (N=1453) and fibrinogen analyses (N=1237) since this is considered evidence of current infection, rather than chronic processes.^15^ Of remaining observations, only 25 participants were missing employment status, but a further 2863 and 2568 participants were excluded due to missing covariates (mostly for body mass index, BMI, occupational social class and General Health Questionnaire (GHQ-12) score, missingness of 5.3%, 3.6% and 3%, respectively, in CRP analyses). The final complete-case sample sizes were 23 025 for CRP models, and 20 724 for fibrinogen models.

### Measures

Current employment status was assessed by questionnaire. Using the International Labour Organisation definition, we considered participants unemployed if they were without work and seeking work, or waiting to take up work.^16^ The baseline group in all analyses was participants in paid employment or self-employment. Participants out of the labour force, due to sickness/disability or otherwise economically inactive (including homemakers, the retired, full-time students, participants in government training or doing unpaid work), were analysed separately. Participants who were unemployed but temporarily prevented from seeking work due to illness were included with the sick/disabled group.

In all surveys, serum CRP concentrations were analysed by the Biochemistry Department of the Royal Victoria Infirmary, Newcastle, using the N Latex CRP mono immunoassay on the Behring Nephelometer II Analyser.^17^ Imprecision at the low end of the analytical range results in a coefficient of variation of <6% for this analyser.^13^ The limit of detection was 0.1 mg/L.

Fibrinogen was analysed at the Royal Victoria Infirmary Haematology Department using a modified Clauss thrombin clotting method. The Organon Teknika MDA 180 analyser was used until HSE 2006^18–21^ when the Auto Coagulation lab (TOP) CTS analyser was introduced.^13^ ^22–25^ A correlation of 0.96 indicates results from the two analysers are comparable.^25^ The limit of detection was 0.2 g. Fibrinogen was not measured for participants taking drugs known to affect fibrinogen. Mean values of CRP and fibrinogen differed between surveys (see online supplementary appendix A).

All covariates except BMI were assessed by questionnaire. Socioeconomic position was indexed by occupational social class (Registrar General's Social Classification) from current or most recent employment, and housing tenure (classified as owns home outright, buying with a mortgage or loan or renting).

Smoking was categorised as never smoker, ex-smoker, current (<10/day), current (10–19/day) and current (20+/day). Alcohol intake was assessed by frequency of drinking occasions in the past year (every couple of months or less, 1–2 times per month, 1–2 times per week, 3–4 times per week, 5+ times per week or never). Height and weight were assessed by the nurse and BMI calculated, with WHO BMI categories (<18.5, 18.5–24.99, 25.0–29.99, 30+) used as measure of adiposity.

Long-term illness (mental or physical) was categorised as none, limiting and non-limiting. Total GHQ-12 score (dichotomised using the standard cut-off of 4+) was included to account for depressive/anxiety symptoms. Non-steroidal anti-inflammatory drugs, systemic corticosteroids, corticosteroid injections, lipid-lowering drugs, β-blockers, diclofenac sodium for gout and aspirin or ibuprofen as an analgesic or antiplatelet were classified as medications that would influence inflammatory marker levels.

### Data analysis

Multivariate linear regression was used to examine associations of unemployment with serum concentrations of CRP and fibrinogen (both log-transformed), and multivariate logistic regression to investigate associations of unemployment with odds of raised CRP, defined as >3 mg/L—the standard cut-off in CRP analyses in recognition of the clinically significant increase in cardiovascular risk past this point.^26^ All analyses used STATA's svyset command to account for clustering in the primary sampling unit.

Country and year were included as covariates, with 2003 (when large numbers of observations were collected in both countries) as baseline. Since only 166 usable observations came from HSE 1999, this was merged with HSE 1998.

Effect modification by gender and age were considered, since studies have indicated associations of unemployment with ill-health may be greater for younger people^1^ and men.^6^ To investigate age modifications, the sample was split into three equal-width age bands, corresponding to early career (16–31), mid-career (32–47) and late-career (48–64) participants. Within each band, age in years was adjusted for.

### Sensitivity analyses

To investigate whether bias could have resulted from conducting a complete-case analysis, we compared age-adjusted, gender-adjusted, country-adjusted and year-adjusted associations between unemployment and inflammatory markers in participants lacking covariate data and other participants. In total 12.7% of the final CRP sample was taking medications with potentially anti-inflammatory effects. To investigate whether their inclusion could have affected results, we compared associations between unemployment and inflammatory markers in these participants and other participants.

## Results

Compared to those excluded, participants retained in final models were older and more likely to be male (both significant p<0.001). The original and final analytic samples are compared in table 1. Age-adjusted associations of inflammatory markers with covariates are shown in online supplementary appendix D.

**Table 1:** Descriptive characteristics of the sample

	Initial sample (16–64, targeted for blood sample) N=49 385	Final analytic sample for CRP N=23 025	Final analytic sample for fibrinogen N=20 724
Age			
Mean (SD)	41.2(13.4)	43.1(12.4)	42.3 (12.2)
	%	%	%
Age group			
16–31 (early career)	26.6	20.1	21.3
32–47 (mid-career)	37.7	40.4	42.2
48–64 (late career)	35.7	39.5	36.5
Gender			
Men	45.0	47.3	47.1
Women	55.0	52.7	52.9
Occupational social class (RGSC) from current or past employment			
i—professional	4.8	5.7	5.8
ii—managerial-technical	28.4	32.2	32.2
iii—nm—skilled non-manual	22.7	23.1	23.3
iii—m—skilled manual	16.7	17.8	17.7
iv—semiskilled manual	16.8	16.5	16.5
v—unskilled manual	5.1	4.8	4.6
Missing	5.5		
Housing tenure			
Owns outright	20.1	22.1	20.9
Buying with a mortgage/loan	52.5	56.3	57.5
Renting/other	27.1	21.6	21.6
Missing	0.3		
Economic status			
In paid employment	68.0	75.2	76.5
Unemployed, seeking work	2.9	2.2	2.2
Sick or disabled	6.2	4.3	3.6
Other economically inactive	22.7	18.3	17.7
Missing	0.3		
Cigarette smoking			
Never smoker	45.2	45.0	45.5
Ex-smoker	25.9	28.9	28.2
Current, <10/day	7.6	7.1	7.1
Current, 10–19/day	11.4	10.6	10.7
Current, 20+/day	8.8	8.4	8.5
Missing	1.0		
Drinking frequency in past 12 months			
Every couple months or less	12.7	11.5	11.3
Once or twice/month	13.2	12.8	12.9
Once or twice/week	31.2	32.5	33.0
3 or 4 days/week	15.8	18.0	18.2
5 days/week or more	16.4	19.0	18.8
Not in past 12 months/non-drinker	10.0	6.2	5.9
Missing	0.7		
BMI categories			
<18.5	1.5	1.1	1.1
18.5–24.99	34.4	38.1	39.4
25–29.99	32.9	39.8	39.9
30+	19.9	21.0	19.6
Missing	11.3		
Limiting long-term illness?			
No long-term illness	61.5	61.7	64.3
Limiting long-term illness	21.2	19.6	18.1
Non-limiting long-term illness	17.2	18.7	17.6
Missing	0.1		
GHQ-12 score			
0–3	80.3	86.3	86.6
4+	14.1	13.7	13.4
Missing	5.6		
Survey			
HSE 1998	25.6	30.6	31.4
HSE 1999	0.6	0.7	0.7
HSE 2003	23.5	23.9	23.9
HSE 2006	22.0	21.1	20.7
HSE 2009	7.1	6.3	6.2
SHeS 2003	12.6	10.9	10.7
SHeS 2008	2.8	2.2	2.1
SHeS 2009	2.9	2.3	2.1
SHeS 2010	2.9	2.2	2.1
Government office region			
Scotland	21.2	17.5	17.0
Northeast	4.5	4.6	4.7
Northwest	11.3	11.9	11.9
Yorkshire and Humberside	8.2	9.0	8.9
West Midlands	8.4	8.7	8.9
East Midlands	7.4	7.9	7.8
East Anglia	8.8	9.1	9.1
London	10.1	8.4	8.4
Southeast	12.5	14.4	14.7
Southwest	7.7	8.5	8.5
Takes medications which could affect inflammation			
Yes	10.5	12.7	7.1
No	89.5	87.3	92.9

CRP, C reactive protein; GHQ-12, General Health Questionnaire; HSE, Health Survey for England; SHeS, Scottish Health Survey.

Unemployment was higher among Scottish participants than English participants at 2.6%, compared to 2.1% in the final CRP sample (table 2). Within England, it was lowest in the Southwest at 1.4%.

**Table 2:** Distribution of employment status (%) in final analytic sample (CRP analyses), by country/region

	All English Surveys	All Scottish Surveys	England: Southwest only
Paid employment	75.6	73.3	74.7
Unemployed	2.1	2.6	1.4
Sick/disabled	4.0	5.8	3.1
Other economically inactive	18.4	18.4	20.7
Total N	18 997	4028	1959

CRP, C reactive protein.

### Unemployment and inflammation

Across the whole sample, log-transformed CRP, log-transformed fibrinogen and odds of CRP >3 mg/L were significantly raised for unemployed, compared to employed participants (table 3). Effects were robust to adjustment for age, gender, socioeconomic position, long-term illness, GHQ-12 score and health behaviours. For all three markers, attenuation occurred with adjustment for SEP (table 3) but additional adjustment made little difference.

**Table 3:** Associations of current unemployment with inflammatory markers: whole-sample analysis

	CRP (mg/L, log-transformed) N=23 025			Fibrinogen (g/L, log-transformed) N=20 724			CRP>3 mg/L N=23 025		
Adjustment level	Coefficient	CI	p Value	Coefficient	CI	p Value	OR	CI	p Value
Age, gender, country, year									
In paid employment	Ref.			Ref.			1.0		
Unemployed	0.22	0.13 to 0.32	<0.001	0.05	0.03 to 0.07	<0.001	1.66	1.35 to 2.03	<0.001
Sick/disabled	0.42	0.35 to 0.49	<0.001	0.07	0.06 to 0.09	<0.001	2.33	2.04 to 2.66	<0.001
Other economically inactive	0.05	0.01 to 0.08	0.01	0.02	0.01 to 0.03	<0.001	1.21	1.12 to 1.31	<0.001
Age, gender, country, year and socioeconomic position									
In paid employment	Ref.								
Unemployed	0.16	0.07 to 0.26	0.001	0.03	0.01 to 0.05	0.001	1.48	1.20 to 1.81	<0.001
Sick/disabled	0.33	0.26 to 0.40	<0.001	0.05	0.03 to 0.07	<0.001	1.97	1.71 to 2.26	<0.001
Other economically inactive	0.04	−0.00 to 0.07	0.07	0.01	0.00 to 0.02	0.01	1.18	1.08 to 1.27	<0.001
Age, gender, country, year, socioeconomic position and long-term illness									
In paid employment	Ref.								
Unemployed	0.15	0.06 to 0.24	0.002	0.03	0.01 to 0.05	0.001	1.45	1.18 to 1.78	<0.001
Sick/disabled	0.21	0.14 to 0.29	<0.001	0.04	0.02 to 0.06	<0.001	1.60	1.37 to 1.85	<0.001
Other economically inactive	0.02	−0.01 to 0.06	0.21	0.01	0.00 to 0.02	0.02	1.15	1.06 to 1.25	0.001
Age, gender, country, year, socioeconomic position, long-term illness and health behaviours									
In paid employment	Ref.								
Unemployed	0.14	0.06 to 0.23	0.001	0.02	0.00 to 0.04	0.02	1.44	1.16 to 1.80	0.001
Sick/disabled	0.18	0.11 to 0.25	<0.001	0.03	0.01 to 0.04	0.003	1.58	1.35 to 1.85	<0.001
Other economically inactive	0.05	0.01 to 0.08	0.01	0.01	0.00 to 0.02	0.003	1.20	1.10 to 1.31	<0.001
Age, gender, country, year, socioeconomic position, long-term illness, health behaviours and GHQ-12									
In paid employment	Ref.								
Unemployed	0.14	0.06 to 0.23	0.001	0.02	0.00 to 0.04	0.02	1.43	1.15 to 1.78	0.001
Sick/disabled	0.18	0.11 to 0.25	<0.001	0.03	0.01 to 0.04	0.002	1.54	1.31 to 1.82	<0.001
Other economically inactive	0.05	0.01 to 0.08	0.01	0.01	0.00 to 0.02	0.003	1.20	1.10 to 1.30	<0.001

CRP, C reactive protein; GHQ-12, General Health Questionnaire.

Significant interactions were found for age band and country, although not for gender. Age-stratified and country-stratified analyses were conducted to investigate further. Within England, interactions of unemployment and government office region were tested for with the Southeast (the largest group) as baseline.

### Stratification by age band, country and region

Associations of unemployment with CRP and fibrinogen were significantly stronger for participants aged 48–64, compared to those aged 16–31 (interaction p=0.004 and p=0.001, respectively). Stratification by age band (table 4) showed that associations with all three markers were strong for those aged 48 and over, but non-significant in the younger groups.

**Table 4:** Fully adjusted* associations of current unemployment with inflammatory markers in whole sample, stratified by age group

Age band	Coefficient/OR	CI	p Value	N (unemployed)	N (total)
16–31					
Log CRP	0.10	−0.06 to 0.26	0.21	188	4621
Log fibrinogen	0.01	−0.02 to 0.05	0.39	177	4411
CRP, dichotomised	1.29	0.86 to 1.95	0.22	188	4621
32–47					
Log CRP	0.07	−0.07 to 0.21	0.34	171	9309
Log fibrinogen	0.00	−0.03 to 0.03	0.99	165	8747
CRP, dichotomised	1.35	0.91 to 2.00	0.14	171	9309
48–64					
**Log CRP**	**0**.**28**	**0**.**13** to **0**.**42**	**<0**.**001**	**146**	**9095**
**Log fibrinogen**	**0**.**07**	**0**.**04** to **0**.**10**	**<0**.**001**	**120**	**7566**
**CRP, dichotomised**	**1**.**57**	**1**.**08** to **2**.**27**	**0**.**02**	**146**	**9095**

The bold text signifies associations in the stratified analyses which are significant at p<0.05.

*Adjusted for age in years, gender, country, survey year, occupational social class, housing tenure, presence of a long-term illness, smoking, alcohol consumption, categorised BMI and dichotomised GHQ-12.

BMI, body mass index; CRP, C reactive protein; GHQ-12, General Health Questionnaire.

Associations with CRP and fibrinogen were considerably stronger for Scottish participants (interactions p<0.001 and p=0.007). Stratification by country (table 5) showed that among English participants, only odds of CRP >3 mg/L was significantly raised for unemployed participants after full adjustment but in Scotland, associations with all three measures of inflammation were robust. Within England, there were significant regional interactions for CRP and fibrinogen (interactions p=0.03 and p=0.02). This was driven by differences in the Southwest, where associations of all three inflammatory markers with unemployment were found to be negative (table 5).

**Table 5:** Fully adjusted* associations of current unemployment with inflammatory markers, all age groups, stratified by country/region

	Coefficient/OR	CI	p Value	N (unemployed)	N (Total)
Scotland					
** Log CRP**	**0**.**43**	**0**.**24** to **0**.**62**	**<0**.**001**	**102**	**4038**
** Log fibrinogen**	**0**.**07**	**0**.**03** to **0**.**12**	**<0**.**001**	**95**	**3522**
** CRP, dichotomised**	**1**.**98**	**1**.**24** to **3**.**17**	**0**.**01**	**102**	**4038**
					
England					
Log CRP	0.07	−0.03 to 0.16	0.18	399	18 997
Log fibrinogen	0.01	−0.01 to 0.03	0.37	365	17 202
**CRP, dichotomised**	**1**.**29**	**1**.**00** to **1**.**66**	**0**.**046**	**399**	**18 997**
ENGLAND—Southwest only					
**Log CRP**	**−0**.**33**	**−0**.**60** to **−0**.**06**	**0**.**02**	**28**	**1959**
**Log fibrinogen**	**−0**.**07**	**−0**.**13** to **−0**.**02**	**0**.**007**	**27**	**1763**
CRP, dichotomised	0.53	0.19 to 1.47	0.22	28	1959

The bold text signifies associations in the stratified analyses which are significant at p<0.05.

*Adjusted for age in years, gender, country, survey year, occupational social class, housing tenure, presence of a long-term illness, smoking, alcohol consumption, categorised BMI and dichotomised GHQ-12.

BMI, body mass index; CRP, C reactive protein; GHQ-12, General Health Questionnaire.

### Sensitivity analyses

Age-adjusted, gender-adjusted, country-adjusted and year-adjusted associations between unemployment and inflammatory markers did not differ between participants lacking covariate data and other participants, indicating their exclusion had not produced bias. Associations did not differ between participants taking anti-inflammatory medicines other participants, indicating their inclusion had not produced bias.

Since the years of data collection differed between the two countries, we considered whether country differences might reflect secular changes in associations of unemployment and health due to the onset of the recession. Analyses were re-run and restricted to 2003, a year well before the recession when large numbers of observations were taken in both countries, but significant country interactions remained for both CRP (p=0.01) and fibrinogen (p=0.05).

To explore whether country/regional differences were due to climate, English observations were stratified into latitudinal bands: The North West, North East and Yorkshire, the Midlands and East Anglia and London and the South. No latitude effect was observed.

In both countries (see online supplementary appendices B and C), attenuation occurred with adjustment for SEP on all measures of inflammation. In contrast, additional adjustment for long-term illness made no difference in either country. Adjustment for health behaviours produced modest attenuation in Scotland, but not for England.

## Discussion

### Unemployment and inflammation

In a large data set representing working-age people in England and Scotland, we found elevations in CRP and fibrinogen among unemployed men and women, compared to their employed counterparts. Results were robust to adjustment for pre-existing illness, social position, health behaviours and symptoms of depression/anxiety. This suggests unemployment is linked to inflammation via pathways independent of these factors and that inflammation may help explain the increased morbidity and mortality repeatedly observed in this group.

Our findings accord with research linking inflammation to social stressors, including bereavement^27^ and caregiving^28^ and disadvantaged socioeconomic position.^29^ ^30^ To our knowledge, two studies have explored associations between unemployment and inflammation. Both were small, with sample sizes of 225^31^and 1227,^32^ and neither carried out in a UK population. Both report that inflammatory markers (CRP and/or Interleukin-6) were raised in unemployed participants compared to working counterparts. Our findings serve to confirm and extend these findings using data from large scale, nationally representative UK studies.

Our results do not support a model whereby the poor health of the unemployed can be explained by direct selection due to poor health. However, in both countries, substantial attenuation occurred with adjustment for SEP, supporting indirect selection by socioeconomic position.

While unemployment is associated with adverse health behaviours,^33^ in our study this did not explain the association of unemployment with raised inflammatory markers. Modest attenuation with adjustment for smoking, drinking and BMI was observed in Scotland, but not in England. This may reflect inaccuracies in measurement of tobacco and alcohol consumption in large-scale health surveys, limiting how effectively these factors can be controlled for. Alternatively, results may support the idea that the relationship of unemployment with health behaviours may itself vary by context.^34^

Associations were largely independent of psychological distress as measured by the GHQ-12. Measurement of psychological distress may not have been optimal in our analyses, since there is more evidence that inflammation is associated with depression than anxiety and the GHQ-12 may be a relatively poor measure of depression. Disadvantaged groups may also tend to under-report symptoms of minor psychiatric disorder as measured by the GHQ,^35^ potentially leading to discrepancies in the accuracy of measurements for biomarkers and psychiatric symptoms.

### Age and country/regional effects

The age modification observed could reflect unemployment being more stressful for older jobseekers, for instance due to outdated skills, or real or perceived job discrimination.^5^ Alternatively, it could reflect accumulation of exposure over the life course. There is substantial evidence that unemployment spells cluster longitudinally within individuals, due to loss of skills or impact on perceived employability.^36^ ^37^ There is also evidence that effects of unemployment on inflammation are lasting and could act additively over time.^32^ Hence, late-career unemployment may be acting as a marker for longer term unemployment and/or more past unemployment, with plausibly greater effects on inflammation.

It is unclear what is driving the country/regional modifications. Sensitivity analyses allowed us to discount differential medication use by country, proximity of data collection to the recession and latitude as explanations. Furthermore, country differences are not consistent with differential selection effects due to variation in background unemployment rate. ‘Direct selection’—the idea that poor health of the unemployed can be largely explained by selection into unemployment of the unhealthy, and/or selection of the healthier unemployed back into employment—predicts weaker associations of unemployment and ill-health in times and places when unemployment is higher. Against a high background unemployment rate, job loss should be less discriminating, selection minimised and the unemployed more ‘normal’ as a result.^2^ Since unemployment benefit rates are determined by central UK government, country effects are unlikely to stem from differential financial impacts of unemployment. Hence, if the differences are not due to any of these processes and persist after full adjustment, results may implicate a genuinely greater impact of unemployment in Scotland via alternative pathways, such as psychosocial stress.

While selection predicts stronger associations of unemployment and ill-health against a low background unemployment rate, there are also theoretical reasons to expect the opposite. It has been suggested that unemployment may be a more stressful experience, with worse effects on health where unemployment is high because jobseekers will perceive prospects for re-employment as worse.^38^ This could produce stronger associations of unemployment and ill-health, despite weaker selection effects.

A final possibility is that country and regional differences may again reflect life course accumulation processes. If unemployment was more widespread in Scotland at the time of data collection and had been during much of these participants’ working lives, then it is likely that unemployed Scottish participants will have been unemployed for longer than their counterparts elsewhere or accumulated more lifetime unemployment, with plausibly greater effects on inflammation. Indeed, this explanation is supported by the stronger associations observed for older participants, since differences stemming from accumulation processes would be expected to emerge later in life.

While this cannot be tested within this cross-sectional data set, support comes from other UK data sources for this period. An analysis of unemployment duration between 1991 and 2006 using the British Household Panel Survey^39^ found probability of re-employment during follow-up was lower in Scotland than in every English region (0.655, compared to the South East).

The negative effects in the South West require a different explanation. Unemployed participants in the South West did not appear different in terms of demographics or health behaviours, but this region had the least unemployment, in accordance with Labour Force Survey data from this period. It is, therefore, likely that these participants will have been unemployed for less time than their counterparts elsewhere, perhaps with better perceptions of re-employment prospects playing an additional protective role. However, these factors cannot explain why inflammatory markers were actually lower for the unemployed compared to employed participants in this region.

Given the small sample sizes in regionally-stratified models, negative effects in the South West could be type 1 errors. Alternatively, differences in three-way selection between the employed, unemployed and economically inactive could be involved. For people with sufficient health problems to claim sickness/disability benefits, the financial incentive to exit the labour market altogether is considerably greater for those who are unemployed than employed, and people do appear to follow these incentives.^40^ Such differential labour market exit would mean that, all else equal, the unemployed should be more selected for *good* health than the employed. Of course, other processes—such as selection of healthy jobseekers back into employment plus any negative causal influences of unemployment on health—would act in the opposite direction, potentially obscuring effects of differential labour market exit. However, in a context of very low unemployment, these effects could plausibly come to the fore, possibly accounting for the negative associations in the South West. If so, effects reported for Scotland, and England overall, should be considered underestimates.

This analysis had several advantages; our sample was much larger than the two previous studies, and contained both men and women from across the working-age range, increasing generalisability of results. By considering a wide range of potential confounders and mediators, we were able to explore confounding by socioeconomic position, by pre-existing illness and the role of health behaviours. Participants who were temporarily sick during a spell of unemployment were excluded, leading to conservative estimates.

This analysis has three main limitations. The first concerns loss of data between those targeted for a blood sample, and the usable CRP and fibrinogen measurements actually obtained; resultant bias cannot be ruled out. Second, comparatively few unemployed women in the sample meant gender modifications could not be fully explored. Third, analysis of current unemployment in the context of life histories was not possible. This would have allowed further exploration of effect modifications by age and region.

## Conclusions

This analysis found robust elevations in CRP, fibrinogen and odds of CRP >3 mg/L among English and Scottish unemployed men and women compared to their employed counterparts, but strength of effects varied considerably by both age and country/region, suggesting the relationship of unemployment with inflammation may be strongly influenced by environmental or contextual factors. Alternatively, if these differences reflect life course accumulation processes, they may indicate long-term or repeated unemployment as especially damaging to aspects of health related to inflammation.

**What is already known on this subject**

- Systemic inflammation is increasingly implicated as a mediating factor relating stress to morbidity and mortality.
- Both morbidity and mortality are elevated during unemployment, but questions remain regarding the direction of causation and mediating mechanisms involved.
- Two small-scale studies have reported elevated inflammatory markers in unemployed participants, consistent with an impact of unemployment on health via psychosocial stress.

**What this study adds**

- We confirm and extend these findings using data from two large-scale, nationally representative studies, and explore this association in a UK context, for the first time.
- While current unemployment was robustly associated with elevated inflammatory markers, effect modifications by both age and region suggest the relationship may be strongly influenced by contextual factors and/or accumulation processes.

## Supplementary Material

Web appendix A

Web appendix B

Web appendix C

Web appendix D
