# Supplementary material for: Elevated inflammatory biomarkers during unemployment: modification by age and country in the UK
Source: J Epidemiol Community Health. 2015 Feb 19;69(7):673–9. doi: 10.1136/jech-2014-204404 (PMC4483793; doi:10.1136/jech-2014-204404)
Supplement: Web appendix A [file jech-2014-204404-s1.pdf]

**APPENDIX A: Inflammatory markers by year and country (final analytic samples)**

| | CRP (mg/L) | | Fibrinogen (g/L) | | CRP>3mg/L | |
|---|---|---|---|---|---|---|
| | N | Mean (S.D.) | N | Mean (S.D.) Fibrinogen | N | % CRP>3mg/L |
| HSE 1998/9 | 7208 | 1.93(2.02) | 6669 | 2.52(0.60) | 7208 | 21.2 |
| HSE 2003 | 5499 | 2.04 (2.07) | 4910 | 2.82(0.64) | 5,499 | 23.5 |
| HSE 2006 | 4847 | 1.97(2.00) | 4268 | 2.86(0.63) | 4847 | 21.9 |
| HSE 2009 | 1443 | 2.00(1.98) | 1277 | 2.98(0.52) | 1443 | 22.8 |
| SHeS 2003 | 2498 | 2.04(2.09) | 2191 | 2.84(0.61) | 2498 | 22.7 |
| SHeS 2008 | 508 | 2.07(1.99) | 431 | 3.13(0.49) | 508 | 24.0 |
| SHeS 2009 | 518 | 2.01 (1.99) | 435 | 3.02(0.59) | 518 | 21.8 |
| SHeS 2010 | 504 | 1.81 (1.84) | 433 | 2.86(0.51) | 504 | 18.3 |
| ALL ENGLISH SURVEYS | 18997 | 1.98( 2.03) | 17202 | 2.73(0.64) | 18997 | 22.2 |
| ALL SCOTTISH SURVEYS | 4028 | 2.01(2.03) | 3522 | 2.90(0.60) | 4028 | 22.2 |
