# Supplementary material for: Elevated inflammatory biomarkers during unemployment: modification by age and country in the UK
Source: J Epidemiol Community Health. 2015 Feb 19;69(7):673–9. doi: 10.1136/jech-2014-204404 (PMC4483793; doi:10.1136/jech-2014-204404)
Supplement: Web appendix B [file jech-2014-204404-s2.pdf]

APPENDIX B: Elevations in inflammatory markers, unemployed vs. employed participants: ENGLAND ONLY									
	CRP (mg/L, log-transformed) N=18997			Fibrinogen (g/L, log-transformed) N=17202			CRP>3mg/L N=18997		
**Adjustment level**	Coeff.	CI	p	Coeff.	CI	p	OR	CI	p
Age, gender, year	0.12	0.02-0.23	0.02	0.03	0.01-0.06	0.004	1.47	1.16-1.86	0.001
+ socioeconomic position	0.07	-0.03-0.18	0.17	0.02	-0.00-0.04	0.12	1.33	1.05-1.69	0.02
+ socioeconomic position and long-term illness	0.06	-0.04-0.17	0.25	0.02	-0.01-0.04	0.15	1.31	1.03-1.66	0.03
+ socioeconomic position, long-term illness and health behaviours	0.06	-0.03-0.16	0.20	0.01	-0.01-0.03	0.39	1.30	1.01-1.67	0.04
+ socioeconomic position, long-term illness, health behaviours and GHQ-12	0.07	-0.03-0.16	0.18	0.01	-0.01-0.03	0.37	1.29	1.00-1.66	<0.05
