# Supplementary material for: Elevated inflammatory biomarkers during unemployment: modification by age and country in the UK
Source: J Epidemiol Community Health. 2015 Feb 19;69(7):673–9. doi: 10.1136/jech-2014-204404 (PMC4483793; doi:10.1136/jech-2014-204404)
Supplement: Web appendix C [file jech-2014-204404-s3.pdf]

APPENDIX C: Elevations in inflammatory markers, unemployed vs. employed participants: SCOTLAND ONLY									
	CRP (mg/L, log-transformed) N=4028			Fibrinogen (g/L, log-transformed) N=3522			CRP>3mg/L N=		
**Adjustment level**	Coeff.	CI	p	Coeff.	CI	p	OR	CI	p
Age, gender, year	0.58	0.40-0.76	<0.001	0.12	0.08-0.16	<0.001	2.49	1.65-3.75	<0.001
+ socioeconomic position	0.49	0.30-0.67	<0.001	0.10	0.06-0.14	<0.001	2.07	1.36-3.13	0.001
+ socioeconomic position and long-term illness	0.48	0.30-0.67	<0.001	0.10	0.06-0.14	<0.001	2.06	1.35-3.14	0.001
+ socioeconomic position, long-term illness and health behaviours	0.43	0.25-0.62	<0.001	0.08	0.03-0.12	<0.001	2.01	1.26-3.21	0.003
+ socioeconomic position, long-term illness, health behaviours and GHQ-12	0.43	0.24-0.62	<0.001	0.07	0.03-0.12	<0.001	1.98	1.24-3.17	0.005
