# Supplementary material for: Elevated inflammatory biomarkers during unemployment: modification by age and country in the UK
Source: J Epidemiol Community Health. 2015 Feb 19;69(7):673–9. doi: 10.1136/jech-2014-204404 (PMC4483793; doi:10.1136/jech-2014-204404)
Supplement: Web appendix D [file jech-2014-204404-s4.pdf]

APPENDIX D: Age-adjusted associations of inflammatory markers with covariates. ◊p<0.05 *p<0.01							
		CRP (mg/L) N=23,025		Fibrinogen (g/L) N=20,724		CRP>3mg/L N=23,025	
		N	Mean	N	Mean	N	% CRP>3mg/L
Age group	16-31 (Early career)	4621	1.69	4411	2.54	4621	18.5
	32-47 (Mid-career)	9309	1.82*	8747	2.71*	9309	19.1
	48-64 (Late career)	9095	2.30*	7566	2.94*	9095	27.1
Age-adjusted estimates for all other covariates: age centred at 41.2		N	Age-adjusted mean	N	Age-adjusted mean	N	Age-adjusted Odds Ratios
Gender	Men	10900	1.79	9759	2.65	10900	1
	Women	12125	2.08*	10965	2.83*	12125	1.45*
Occupational social class (RGSC) from current or past employment	i -professional	1300	1.65	1198	2.65	1300	1
	ii-managerial-technical	7412	1.81*	6666	2.71*	7412	1.28*
	iii-nm - skilled non-manual	5309	2.02*	4818	2.77*	5309	1.63*
	iii-m - skilled manual	4100	1.99*	3664	2.72*	4100	1.51*
	iv - semi-skilled manual	3808	2.10*	3416	2.80*	3808	1.75*
	V – unskilled manual	1096	2.08*	962	2.79*	1096	1.62*
Housing tenure	Owns outright	5083	1.79	4331	2.73	5083	1
	Buying with a mortgage/loan	12959	1.87◊	1,924	2.71	12959	1.09
	Renting/other	4983	2.26*	4469	2.84*	4983	1.64*
Cigarette smoking	Never smoker	10365	1.79	9422	2.69	10365	1
	Ex-smoker	6660	1.90*	5852	2.68	6660	1.07
	Current, <10/day	1629	1.96*	1477	2.79*	1629	1.22◊
	Current, 10-19/day	2436	2.23*	2207	2.91*	2436	1.51*
	Current, 20+/day	1935	2.51*	1766	2.97*	1935	1.91*
Drinking frequency in past 12 months	Every couple months or less	2654	2.27	2331	2.94	2654	1
	Once or twice/month	2951	2.05*	2674	2.89*	2951	0.83*
	Once or twice/week	7474	1.96*	6828	2.76*	7474	0.72*
	3 or 4 days/week	4136	1.75*	3768	2.65*	4136	0.59*
	5 days/week or more	4376	1.74*	3902	2.56*	4376	0.59*
	not in last 12 months/non-drinker	1434	2.17	1221	2.88*	1434	0.88
BMI categories	18.5-24.99	8778	1.40	8166	2.62	8778	1
	25-29.99	9174	1.90*	8276	2.74*	9174	1.70*
	30+	4829	3.12*	4060	3.00*	4829	4.91*
	<18.5	244	1.22	222	2.57	244	0.87

Limiting long-term illness?	No long-term illness	14216	1.80	13321	2.72	14216	1
	Limiting long-term illness	4502	2.33*	3759	2.81*	4502	1.66*
	Non-limiting long-term illness	4307	2.03*	3644	2.76*	4307	1.27*
GHQ-12 score	0-3	19876	1.90	17955	2.73	19876	1
	4+	3149	2.17*	2769	2.79*	3149	1.36*
Currently taking NSAIDs, statins, corticosteroids, beta-blockers or antidepressants	No	20094	1.88	19256	2.74	20094	1
	Yes	2931	2.44*	1468	2.78◊	2931	1.64*
